# Worms like catnip too! Identification of a new odor attractant in *C. elegans*

**DOI:** 10.17912/micropub.biology.000568

**Published:** 2022-05-06

**Authors:** Brianna Ramos, Bryant Cruz, Alec DeLong, Gianina Pontrelli, Gareth Harris

**Affiliations:** 1 California State University Channel Islands, Biology program, Camarillo, Ca, USA; 2 Neuroscience Graduate Program, University of Michigan, Michigan, USA

## Abstract

Organisms across the phyla are capable of sensing an array of sensory cues to control, or shape behavioral responses in order to survive in a complex environment consisting of an array of attractive and repulsive dangerous cues. Mammalian systems extensively use olfactory and gustatory behavior to fine tune sensory-dependent decision-making behaviors. Despite understanding the importance of behavioral responses to cues in the form of odors in shaping decision-making behavior. The underlying mechanisms that mediate these responses at the level of sensation, processing, integration, and modulation of these sensory dependent responses are not fully understood. To understand these mechanisms we use the invertebrate worm, *C. elegans,* to characterize attraction to mammalian sensed odorant cues. We show that hermaphrodite worms are attracted to catnip oil cues, and identify select sensory mechanisms that mediate this attraction, identifying multiple sensory genes that are involved in this chemosensory response to a sensed cue, that is highly attractive in many cats. We have identified sensory transduction mechanisms, including G-proteins and cyclic nucleotide-gated ion channels, that regulate odor-dependent attraction to mammalian sensed catnip oil cues. We therefore provide a platform to use *C. elegans* as a model for studying olfactory-dependent pathways to mammalian cues. This allows characterization of the neural mechanisms that shape olfactory behavior and decision-making in higher systems.

**Figure 1. Worms perform odor guided attraction to catnip oil cues and this requires specific sensory transduction genes:**
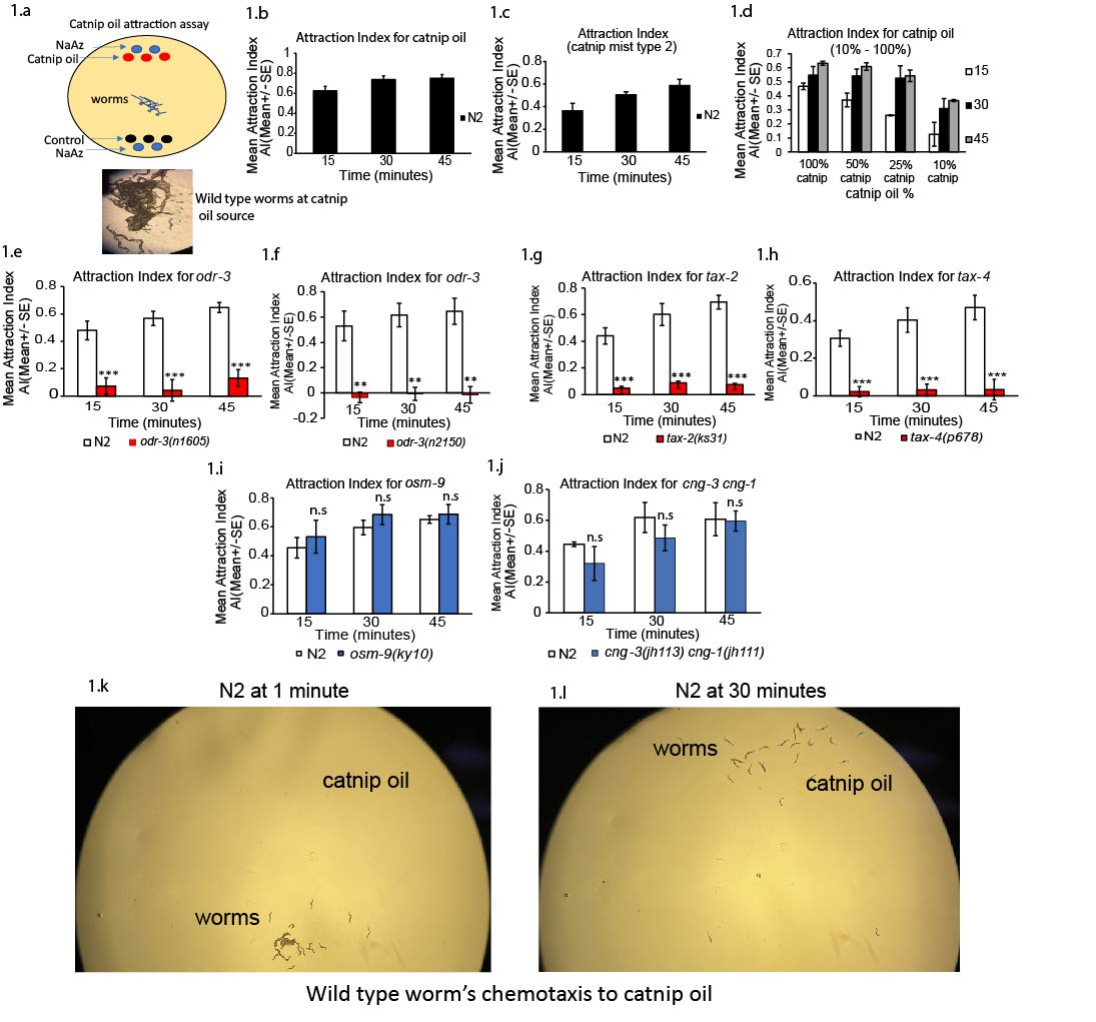
a) **Catnip oil attraction can be measured using a chemotaxis population assay** . Schematic of behavioral assay for catnip oil attraction shown. Adult hermaphrodite worms used for all attraction assays indicated in schematic diagram and in above figure (1a - 1l). b) **Graph showing catnip oil attraction index across 15 minute intervals for wild type adult worms.** Worms were analyzed across 0 - 45 minutes (at 15, 30 and 45 minute time points for attraction to catnip oil source) after catnip oil odor presentation (catnip oil sample 1). This involved counting worms at catnip oil, control and on rest of assay plate to determine attraction index at 15, 30 and 45 minutes after catnip oil addition. (n=6 repeated days). Black bars show positive attraction Index across 45 minute assay. c) **Catnip oil sample 2 repeating attraction behavior** (additional catnip oil, Brand-Catnip mist product), wild type attraction observed (n=6 repeated days), experiment shows 0-45 minutes after catnip oil exposure. Black bars show positive Index across 45 minute assay. d) **Catnip oil shows an overall chemotaxis across multiple dilutions.** Worms were examined for attraction at 10%, 25%, 50% and 100% catnip oil (n=2 repeated days), experiment shows 0 - 45 minutes after catnip oil exposure. e-f) **Removal of sensory neuron expressed G-protein alpha subunit, ODR-3, disrupts attraction to catnip oil.** Animals lacking *odr-3* -encoded G-protein alpha subunit resulted in a reduced attraction Index to catnip oil. *odr-3(n1605)* (n=8 repeated days), and *odr-3(n2150)* (n=4 repeated days), respectively. *e) odr-3(n1605)* and *f) odr-3(n2150)* were compared to wild type N2 animals, experiment shows 0-45 minutes after catnip oil exposure. White bars = wild type N2 animals, red bars = mutant animals. g-h) **Removal of cGMP-gated ion channel subunits, TAX-2 and TAX-4, reduces catnip oil attraction.** Animals lacking. g) *tax-2* and h) *tax-4* -encoded cation channels (cGMP-gated channel) abolish attraction to catnip oil when compared to wild type N2 animals. n=7 repeated days examined for *tax-2(ks31)* mutants, and n=6 repeated days tested for *tax-4(p678)* mutants shown, experiment shows 0 - 45 minutes after catnip oil exposure. White bars = wild type N2 animals, red bars = mutant animals. i) **Removal of TRPV related-channel, OSM-9 has no effect on catnip oil attraction.** *osm-9(ky10)* animals. *osm-9* mutants were compared to wild type N2 animals tested in parallel, experiment shows 0-45 minutes after catnip oil exposure. n=5 repeated days. White bars = wild type N2 animals, blue bars = mutant animals. J) **Removal of cyclic nucleotide-gated channels, *cng-3* and *cng-1* ( *cng-3 cng-1* double mutant) has no effect on catnip oil attraction.** Compared to wild type hermaphrodites tested in parallel, experiment shows 0-45 minutes after catnip oil exposure. n=3 repeated days tested for *cng-3 cng-1* double mutants compared to wild type N2 animals. White bars = wild type N2 animals, blue bars = mutant animals. K-l) **Image showing chemotaxis to catnip oil across 30 minutes (k) At 1 minutes. (l) at 30 minutes after catnip oil addition.** Wild type N2 animals shown only in image k and l after 1 and 30 minutes of exposure to catnip oil. Wild type animals show chemotaxis towards attractive catnip oil source. Chemotaxis assay for wild type N2 worms across 0–45 minutes. All worms were washed and prepared as shown in methods and tested as young adult hermaphrodite animals. Mean +/-SE, *Students T Test* was performed to compare mutants to parallel tested wild type N2 worms tested on the same day. Student’s *t* test, * p ≤0.05, ** p ≤0.01, *** p ≤0.001, n.s., not significant. n=number of repeated days tested in A-J. Each day of experiments contained between 50-100 wild type worms and mutant worms per assay.

## Description

An organism’s behavior and decision-making can be influenced by olfactory behavior. Animals across the phyla constantly utilize chemosensory functions in order for survival (Ache and Young, 2005; Chaisson and Hallem, 2012; Lessing and Carlson, 1999). Organisms are also able to couple odor sensation with physiological responses and behavioral states to coordinate specific behavioral responses involved in bonding, social interaction, mating and feeding. For example, mammals, such as, felines/cats respond to chemicals in the form of odors, using olfaction and respond based on processing of these types of cues at multiple levels of the brain to regulate behavior (Hart *et al* ., 1985, Miyazaki *et al* ., 2018, Jacinto *et al.* , 2019). Despite understanding these important strategies, the neural molecules and circuit’s underlying these behaviors are not fully understood. One fascinating behavioral response to chemical stimuli is the behavior of many cats to catnip. The plant terpenoid, nepetalactone is one of the main chemical constituents of the essential oil of *Nepeta cataria* (McElvain *et al* ., 1941, Tucker and Tucker, 1988, Bol *et al* ., 2017 *)* . This is thought to attract a large percentage of cats based on odor-guided behavior mediated through the olfactory system, and produce overall a euphoric and hallucinogenic effect (Hart *et al* ., 1985, Wolski *et al* ., 1980, Ellis and Wells, 2010). Catnip is also sensed and produces distinct behavior in additional organisms, including, fruit flies and Mosquito's (Bernier *et al* ., 2005, Shi *et al* ., 2021, Melo *et al* ., 2021).

We use the invertebrate nematode, *Caeanorhabditis elegans* to understand behavioral responses to the cat attractant, catnip oil. *C. elegans* are able to respond to an array of attractants and repulsive odor cues, using a variety of sensory neurons, and distinct sensory transduction pathways (Ferkey *et al* ., 2021, Ghosh *et al* ., 2017, Bargmann *et al* ., 1993, Bargmann, Wormbook, Chemosensation, 2006). We have provided a platform to study attractive behavior toward a known mammalian sensed odor cue, catnip oil, based on chemotaxis to catnip oil being observed in *C. elegans* . Using a chemotaxis assay we have shown that worms chemotaxis toward catnip oil (Hart *et al* ., 1985, Bargmann and Horvitz, 1991, Bargmann *et al* ., 1993, Troemel *et al* ., 1997). We have used this behavioral paradigm to examine responses in *C. elegans* to catnip oil, and begin to dissect the chemosensory mechanisms that allow coordinated attraction to catnip oil. We have used a mutant analysis approach to characterize any role of sensory mechanisms in any odor-guided attraction to catnip oil. We hope this work will provide insight and a platform into studying the neural mechanisms and signals that drive olfactory behavior to mammalian sensed odor attractants and repellents, sensed by mammals, such as cats.

To investigate the behavioral response towards catnip oil, we examined chemotactic behavior toward catnip oil stimuli(catnip mist product). To do this we used a previously described chemotaxis assay with modifications (as described in, Bargmann and Horvitz, 1991, Bargmann *et al* ., 1993). We examined the ability of *C. elegans* to respond to the catnip oil. We examined chemotaxis of the wild type worm towards catnip oil at different time points (15, 30, and 45 minutes) (See Figure 1a-b, materials and methods). Catnip oil stimuli originating from the catnip mist spray was used directly and undiluted for all chemotaxis assays unless otherwise stated (See materials and methods).

We examined young adult wild type animals for their chemotaxis to/or away from the catnip oil point source (See schematic-Figure 1a). Interestingly, we observed a strong attraction to this catnip oil stimuli (Figure 1a-b, Figure 1k-l for images of wild type N2 worms in assay). Wild type animals across 45 minutes performed attractive behavior to catnip oil, as seen in Figure 1(b) based on positive attraction index observed, ranging from +0.5 to +0.7 index (Figure 1b, images seen shows worms at the catnip oil source during catnip oil chemotaxis, 1k-l). This suggests that *C. elegans* is attracted to the odors generated by catnip oil during this long distant chemotaxis assay. To determine the sensitivity of the wild type hermaphrodites to the catnip oil, we diluted the catnip oil to concentrations to 50%, 25%, and 10% of the total original catnip oil stimuli (Figure 1d, see methods). We examined wild type worms at various concentrations and found that worms chemotax to the catnip oil stimuli between 10% – 100% (Figure 1d). Although the concentration of 100% and 50% catnip oil was overall stronger than 25%, and weaker at 10% catnip oil stimuli (Figure 1d). We also tested an additional catnip oil product to show this response is consistent across multiple catnip oil products (Figure 1c). This also showed chemotaxis behavior to the catnip oil stimuli (Figure 1c). Supporting, that wild type N2 hermaphrodite worms are sensitive to catnip oil and produce an attraction across different catnip oil attractants that we have used for this present study.

Based on this attractive behavior to a cat sensed attractant, we sought to dissect the genes and neuronal pathways that drive chemosensory behavior and location of catnip oil stimuli. In this study, we performed a time course experiment across 45 minutes, that included multiple time points (15, 30, 45 minutes, Figure 1e-j) analyzing chemoattraction in various sensory transduction mutants, to begin to characterize chemotaxis towards catnip oil.

We first sought to identify the signal transduction pathways responsible for catnip oil attraction (Figure 1). We examined mutants lacking significant portions of sensory transduction across the amphidal sensory neurons. We examined animals lacking G-protein alpha subunits and identified mutants that lack G-protein alpha signaling expressed in chemosensory neurons (Figure 1, Jaansen *et al* ., 1999; Royaie *et al* ., 1998) (Figure 1e-1f). Interestingly, animals lacking the G-protein alpha subunit, *odr-3,* through examining two loss of function mutants, *odr-3* ( *n2150* ) and *odr-* 3( *n1605* ) were both defective for chemotaxis to catnip oil (Figure 1e-1f, Royaie, *et al* ., 1998). This suggests that *odr-3-* encoded G-protein alpha subunits are important for catnip oil attraction.

We also examined various mutants lacking a number of ion channels expressed in sensory neurons that have been implicated in various sensory functions (Coburn and Bargmann, 1996; Tobin *et al* ., 2002; Harris *et al* ., 2014). The *C. elegans* nociceptive-like TRPV channels (OSM-9), cyclic nucleotide (cGMP)-gated ion channels (TAX-2/4) and cyclic-nucleotide gated channel subunits (CNG-1/CNG-3) (Figure 1g-j; Cho *et al* ., 2004; He *et al* ., 2016; Coburn and Bargmann, 1996; Komatsu, *et al* ., 1996; Tobin *et al* ., 2002). Interestingly, *osm-9* mutants ( *osm-9(ky10* )) lacking the TRPV related channel has no effect on catnip oil attractive responses, suggesting no obvious role for *osm-9* in this odor attraction. In contrast, mutants lacking *tax-2* gene function exhibited severely compromised olfactory behavior to catnip oil (evident by low attraction index and minimal worms at the catnip oil) (Figure 1g). *tax-2(ks31)* loss of function mutants exhibited defective catnip oil chemotaxis (Figure 1g, Coburn and Bargmann, 1996). Finally, we examined *tax-4* null mutants ( *tax-4(p678)* ) that lack a cGMP-gated channel subunit also show a significant reduction in chemotaxis to catnip oil (Figure 1h). In addition, examination of mutants lacking *cng-3* and *cng-1* ( *cng-3(jh113) cng-1(jh111)* mutants) shows normal catnip oil chemotaxis (Figure 1j). This suggests that *odr-3* -encoded G-protein alpha subunits, and cGMP-gated channels, encoded by *tax-2* and *tax-4* are required for attractive behavior to catnip oil, but not *osm-9* -encoded TRP-related channels or *cng-1/3-* encoded CNG channel subunits. Suggesting, we have identified an additional mammalian sensed odor cue that in this case attracts *C. elegans* , and have begun to characterize the neural mechanisms driving this chemosensory behavior.

## Methods

**The following strains were tested in the present study.**

Strains were grown in standard conditions. Mutant animals analyzed: Wild type N2(Bristol strain), CX10 *osm-9(ky10);* FK104 *tax-2(ks31),* PR678 *tax-4(p678),* CX2205 *odr-3(n2150),* CX3222 *odr-3(n1605),* KJ5560 *cng-3(jh113) cng-1(jh111).* All mutant strains were examined and compared to wild type animals in our present study.

**Methods**

Wild type worms were grown as previously described (Brenner *et al* ., 1974; Hart *.* , Wormbook, 2006). Wild type worms were cultivated on *E. coli* OP50 standard lab food source prior to testing in attraction assays. Wild type worms (N2) Bristol worms (CGC, Minnesota) will be used in all chemotaxis assays and compared to all mutants tested in parallel. 6 cm (Thermofisher) NGM agar plates were used for behavioral assays with no food present on the assay plate. All wild type animals were grown at 21-23 degrees with sufficient *E. coli* OP50 food source prior to examination of all worms. All wild type worms tested in this study were young adult synchronized hermaphrodite worms under well-fed/non-starved conditions.

**Catnip oil attraction assays**

Chemotaxis assays plates are NGM(Nematode Growth Media) prepared 2 days before each assay. Approximately 50 – 100 wild type young adult worms were tested in each population assay. Briefly, NGM plates used for the assay were left at room temperature on the day of the behavioral assay. Wild type worms are washed 3 times in S. basal solution (As described in Bargmann *et al* ., 1993, Bargmann and Horvitz, 1991; Troemel *et al* ., 1997). The remaining worm pellet was added through pipetting to the center of the NGM assay plate as seen in figure 1a. Once the worm pellet had dried (5 minutes), 3 drops of 1 microliter catnip oil, 1 microliter per drop (Smarty Cat-Catnip Mist Spray, contains catnip oil(nepetalactone), emulsifier and water, Petco.com, Worldwide.inc, Ca) (McElvain *et al* ., 1941, Tucker and Tucker, 1988) was added at one side of the chemotaxis plate (as shown in Figure 1, schematic diagram). A control sample of S. basal or NGM was added to the opposite side of the assay plate. Small drops of sodium azide (1 microliter at 1M) were used to paralyze worms either at the catnip oil source or control source (1 microliter). Worms that are located in the center of the plate at the beginning will be left to analyze their chemotaxis over multiple time points (15, 30, and 45 minutes after worms were added to assay plate), which will either be analyzed for attraction, repulsion or no biased to the catnip oil stimuli. To determine any chemotactic attraction or avoidance, index's were generated based on calculating distribution of worms across NGM agar assay plates at 15, 30, and 45 minutes after catnip oil addition (see Figure 1a-l). To determine attraction index throughout the assay with wild type and all mutants (Bargmann *et al* ., 1993). Total number (#) of worms at odor source (paralyzed at catnip oil)-total number(#) of worms paralyzed at control sample on opposite side of the assay plate/total number(#) worms on the whole chemotaxis assay plate counted at the beginning of the assay (i.e. includes all worms at attractant, control and worms that did not make either point source). Attraction Index values range from 0 to +1.0, 0 being no attraction and increasing positive number representing stronger attraction (Bargmann *et al* ., 1993 and Wormbook.org). For 1e-1j, all catnip samples used for chemotaxis were undiluted product from original Catnip Mist Spray sample 1.

For analysis of dilutions of catnip oil, catnip oil mist was added to an S. basal solution in a 15mL falcon tube, mixed thoroughly and immediately then 1 microliter volumes were added to the chemotaxis assay plates to achieve 10% diluted, 25%, 50% or undiluted catnip oil sample from original catnip oil bottle (See Figure 1d). For the second sample(Spray sample 2) of catnip oil used(Catnip Spray Mist, Leaps & Bounds, contains natural catnip oil, Petco.com, San Diego). For both Catnip Mist products, both contained catnip oil used in this present study.

All mutant animals were compared to wild type animals tested in parallel on the same day. Mean+/-SE, was determine per experiment and a *Students T Test* was performed for examination of significant differences between wild type and mutant genotype's at each time point (15, 30 and 45 minutes), tested on same day. Across all mutants tested (n) = number of repeated days tested in A-J, ranges from 4-8 repeated days tested per mutant genotype.

## Reagents

First catnip oil sample: "Catnip oil mist"(containing catnip oil), Worldwide inc, San Rafael, Ca. Petco.com.

Second catnip oil sample: catnip oil used(Catnip Spray Mist, Leaps & Bounds, contains natural catnip oil, Petco.com, San Diego).
